# Evaluation of *Salvia hispanica* as a Therapeutic Agent against Sodium Arsenic-Induced Testicular Toxicity in a Male Rats Model

**DOI:** 10.3390/life14010109

**Published:** 2024-01-10

**Authors:** Sara Mahmoud Omar, Nasser Nesim Zahran, Rashed A. Alhotan, Elsayed Osman Hussein, Branislav Galik, Ahmed Ali Saleh

**Affiliations:** 1 Nutrition and Food Science Department, Faculty of Home Economics, AL-Azhar University, Tanta 31732, Egypt; saraomer@he.azu.edu.eg; 2 Department of Therapeutic Nutrition, Menoufia University Hospitals, Shebin El-Kom 11352, Egypt; mohamed.mahran@hec.menofia.edu.eg; 3 Department of Animal Production, College of Food & Agriculture Sciences, King Saud University, P.O. Box 2460, Riyadh 11451, Saudi Arabia; ralhotan@ksu.edu.sa (R.A.A.); elsayedosman@khu.org.sa (E.O.H.); 4 Institute of Nutrition and Genomics, Slovak University of Agriculture in Nitra, Slovakia. Trieda A. Hlinku 2, 94976 Nitra, Slovakia; branislav.galik@uniag.sk; 5 Department of Poultry Production, Faculty of Agriculture, Kafrelsheikh University, Kafr El-Sheikh 333516, Egypt

**Keywords:** thyroid hormones, sodium arsenite, sperm parameters, *Salvia hispanica*, testicular toxicity, LH, FSH, testosterone and antioxidant enzymes

## Abstract

Chia seeds offer therapeutic properties that aid in the prevention of a variety of ailments, including cardiovascular disease, diabetes, obesity, and other risk factors. Arsenite, a common environmental chemical, has been identified as a reproductive toxin owing to its negative effects on male reproductive health. It has been shown to inhibit spermatogenesis and generate androgenic effects in men. The primary goal of this research was to look into the effect of *Salvia hispanica* on testicular toxicity caused by sodium arsenite in male rats. A set of 36 male albino rats was allocated to a negative control cohort. The individuals in this group were given a basic meal and orally given distilled water for a duration of 28 days. The other five groups were given a regular meal and received intra-peritoneal injections of sodium arsenite (NaAsO_2_) at a concentration of 4 mg/kg body weight that was diluted in a 0.9% NaCl solution. The injections were administered consecutively, with two doses given within a two-day period. Subsequently, the rats were categorized into several groups using the following classification: Group 2 consisted of a positive control cohort, in which the rats were given a typical baseline diet. Groups 3, 4, 5, and 6 were given a basic diet that included varying proportions of ground chia seeds, namely 5%, 10%, 15%, and 20% per 100 g of the diet. After the trial was completed, the rats were euthanized, and further biological examination was conducted. The measurements of the reproductive organs were documented and reported. The research assessed the following characteristics: sperm count, motility, progressive motility, and normal morphology. The research included examining serum sex hormones, namely luteinizing hormone (LH), follicle-stimulating hormone (FSH), and testosterone. An evaluation of the activity of antioxidant enzymes was performed in the tissue of the testicles. There were statistically significant improvements in the sperm parameters, serum sex hormone levels, and the activity of antioxidant enzymes, such as GPX, SOD, and CAT, in the therapy groups. The levels of malondialdehyde (MDA) exhibited a noteworthy decrease (*p* ≤ 0.05) when compared to the positive control group. *Salvia hispanica* seeds have demonstrated a significant level of effectiveness in reducing sodium arsenite-induced testicular toxicity, which leads to the conclusion. The flavonoid content and antioxidant properties of *Salvia hispanica* seeds may be to blame for the observed behavior. These indicated characteristics may have therapeutic significance in treating testicular harm induced by arsenite exposure.

## 1. Introduction

Currently, people are regularly exposed to a wide range of environmental pollutants that come from both natural and human-caused sources [1]. Specific dangerous compounds have the capacity to act as endocrine disruptors, resulting in diverse health implications. These drugs may interfere with the production, release, and breakdown of hormones that are crucial for both the growth and balance of the body [2].

Arsenic, a substance known to cause cancer, has detrimental effects on the health of humans, animals, and plants by interfering with their physiological processes, growth, and development. It is present in both inorganic and organic forms, and plants’ absorption and accumulation of the substance affects its toxicity. Growing crops on contaminated soil increases the risk of human poisoning [3]. The main pathway by which humans are exposed to arsenic is through the use of polluted drinking water, which mostly contains inorganic forms of arsenic. Human exposure to arsenic mostly occurs via the ingestion of water, food, and pharmaceuticals. Additionally, arsenic may be found in herbicides, insecticides, rodenticides, and food preservatives [4]. The increase in levels of arsenic in the atmosphere has become a global issue due to its harmful effects on the health of both humans and animals [5].

The metalloid in issue has been discovered to have deleterious effects on the well-being of several creatures, including cattle, wildlife, and humans. The consequences of this situation are evident in the form of damage to the gastrointestinal system, toxicity in the blood, and impaired brain function [6]. Researchers have already found a link between being exposed to inorganic arsenic for a long time and metabolic disorders, cancers, problems with the endocrine system, and issues with reproduction [7]. Arsenic exposure has adverse effects on the reproductive traits of young male rats before reaching sexual maturity, potentially resulting in reproductive disorders such as impotence, sperm damage, and prostate cancer. It interferes with the production of sperm and reduces the capacity of sperm to move, the number of sperm, and their ability to survive in the epididymis, possibly affecting male fertility throughout adulthood [8]. As per reference [9], exposure to arsenic (As) has been shown to have adverse effects on certain male reproductive parameters. The repercussions include a decline in the quality of semen, a fall in hormone levels in the bloodstream, and the manifestation of abnormalities in the testicular tissue.

A scientific study by [10] suggests that adult females who are exposed to arsenic have alterations in the morphology of their reproductive organs, the duration of their estrous cycle, the concentrations of sexual hormones, and the functioning of antioxidant enzymes in their uterus and ovaries. A lot of research has shown that, when inorganic arsenic breaks down inside cells, it creates reactive oxygen species and reactive nitrogen species, which cause oxidative and nitrogen stress [11]. Also, it is known that arsenic can throw off the balance of hormones because it binds very strongly to the estrogen receptor α, as shown in [5]. Adequate consumption of nutrients is essential in reducing the likelihood of developing various illnesses linked to the existence of toxic metals, such as arsenic. Both governmental entities and non-governmental groups provide dietary guidance with the aim of safeguarding human health [12]. The importance of bioactive food components, including polyphenols, carotenoids, phytoestrogens, sterols, vitamins, dietary fiber, fatty acids, probiotics, prebiotics, and bioactive peptides, in promoting health is increasingly being acknowledged [13].

*Salvia hispanica*, often known as chia seeds, is an herbaceous plant species belonging to the *Lamiaceae* family [14]. The ingestion of this drug has been recorded as both a dietary and medicinal procedure. The seeds contain a significant amount of protein, essential fatty acids, dietary fiber, antioxidants, flavonoids, anthocyanins, vitamins, carotenoids, minerals, and lipids, especially omega-3 fatty acids. Omega-3 fatty acids are important for the emulsification and absorption of lipid-soluble vitamins [15]. According to [16], *Salvia hispanica* is a significant source of both soluble and insoluble dietary fibers, and it also has antioxidant and anti-inflammatory properties. Chia seeds have a higher protein level, ranging from 19% to 23% compared to other grains, including wheat (about 14%), maize (about 14%), rice (roughly 8.5%), oats (~15.3%), barley (~9.2%), and amaranth (~14.8%) [17].

Seeds from *Salvia hispanica* are the primary raw material acquired on a large scale in industrial settings. Chia seeds are very nutritious, primarily because they contain significant amounts of polyunsaturated fatty acids (PUFA) and dietary fiber. Furthermore, the seeds are rich in nourishing protein and other phenolic compounds, as well as essential macro- and microelements and vitamins [18]. The weight of *Salvia hispanica* seeds is composed of carbohydrates, which make about 26–41% of their total weight. The seeds have a dietary fiber content of around 30–34%. This fiber is composed of plant carbohydrate polymers, including oligosaccharides and polysaccharides such as cellulose, hemicelluloses, pectic compounds, and gums. These components may be linked to lignin and other non-carbohydrate elements. The chia seeds contain around 85–93% insoluble fiber and 7–15% soluble fiber [19]. *Salvia hispanica* seeds consist of 15–25% protein. Globulin is the primary storage protein in chia seeds, constituting 52% of the total protein composition. Albumins, glutelins, and prolamins are proteins that are present in smaller quantities, accounting for 17%, 14%, and 12%, respectively. There are many different kinds of polyphenols in *Salvia hispanica* seeds. These include phenolic acids (like gallic acid, caffeic acid, ferulic acid, and p-coumaric acid), depsides (like chlorogenic acid and rosmarinic acid), flavonoids (like apigenin, kaempferol, quercetin, myricetin, and rutoside), isoflavones (like daidzein, glycitin, genistein, and genistin), and catechin derivatives (like epicatechin) [20]. Chia seeds also contain trace levels of the following sterols: campesterol (472 mg/kg), stigmasterol (1248 mg/kg), β-sitosterol (2057 mg/kg), and ∆5-avenasterol (355 mg/kg). In addition, *Salvia hispanica* seeds contain a substantial quantity of vital bioelements. The elements that are essential for plants may be divided into two categories: macroelements and microelements. Macroelements include phosphorus (P), iron (Fe), manganese (Mn), calcium (Ca), potassium (K), magnesium (Mg), sodium (Na), and sulfur (S). Microelements include zinc (Zn), copper (Cu), molybdenum (Mo), and selenium (Se). The seeds of *Salvia hispanica* are rich in vitamins, namely B-complex, C, A, and E [21].

Chia seeds have beneficial characteristics that help prevent several illnesses, including cardiovascular disease, diabetes, obesity, and related risk factors [22]. Scientific data confirms that chia has immune-enhancing qualities and may be used therapeutically to manage illnesses such as anti-inflammatory disorders, diabetes, hypertension, and dyslipidemia. The importance of this chemical is evident in its potential advantages in terms of improving eyesight, preventing blood clotting, promoting regular bowel movements, alleviating symptoms of depression, reducing anxiety, and providing pain relief; it also possesses antioxidant characteristics [23].

The primary objective of this research is to investigate the potential benefits of *Salvia hispanica* seeds powder, often referred to as chia seeds, on male albino rats. Specifically, we want to examine its anti-inflammatory and antioxidant characteristics, as well as its content of flavonoids, anthocyanins, vitamins, and essential minerals. The main aim of this research is to evaluate the preventive effects of chia seeds on testicular abnormalities induced by exposure to arsenic.

## 2. Materials and Methods

This study followed regulations established by Kafrelsheikh University in Egypt (Number 4/2016 EC) and approved by the local experimental animal care ethics committee.

### 2.1. Materials

- The chia seeds, scientifically known as *Salvia hispanica*, were obtained from the Local Company for Herbs and Medicinal Plants located in Cairo Governorate, Egypt.
- The powder form of sodium arsenite (NaAsO_2_) was acquired from Al-Gomhoria Company for Chemicals, located in Cairo, Egypt.
- The casein, which contains 85% protein as well as choline chloride, DL-methionine, vitamins, and a salt mixture, was procured from El-Fagr Company, located in Cairo, Egypt. Sunflower oil and maize starch were sourced from the local market in Tanta, Egypt.
  ▪ A basal diet is a diet that provides the necessary amount of calories to produce basal heat as well as sufficient critical elements to fulfill fundamental requirements. Basal diet studies use a methodology in which the effects of a certain ingredient, which has yet to be identified, are first excluded from the diet and then introduced.
  ▪ A standard diet refers to a typical diet that does not impose any dietary limitations. An ideal diet should include the whole spectrum of attributes associated with good health, namely: balance, nutritional adequacy, moderation in food consumption, diversity in food choices, and careful calorie management.
  ▪ A baseline diet is essential for all animals before considering any supplements or unconventional dietary approaches.
- A total of 36 male albino rats of the “Sprague Dawley” strain, with an average weight of 200 ± 10 g, were procured from the experimental animal colony maintained by the Ministry of Health and Population in Helwan, Cairo, Egypt.

### 2.2. Methods

(a) The chia (*Salvia hispanica*) seeds were subjected to a washing process using tap water in order to eliminate any extraneous matter. The chia seeds were measured using a laboratory electronic analytical balance (Explorer Pro, Swiss Company, Bern, Switzerland). The chia seeds were processed and placed in low-density polyethylene (LDPE) plastic bags with a wall thickness ranging from 30 to 60 microns. The chia seeds were dehydrated in a WTB binder Model 78,532 oven from Germany at a temperature of 63 °C for a duration of 30 min, using the low-temperature long time (LTLT) method. The drying trays were filled with chia seeds to a depth of 25 mm and then put in the oven at a room temperature of 26 °C. The duration required to obtain the treatment temperature of 63 °C was 11 min. The dried chia seeds were pulverized using a laboratory blender (Waring Commercial, manufactured in the New York, NY, United States), then filtered through a 0.2 mm screen and sealed in a vacuum-packed container. The powder was stored at a temperature of 5 °C until it was ready for examination [24]. The chia seed was milled using a grinder and measured according to three groups of particle size ranges: 200−600 μm (grinding time of 10 s), 100−400 μm (grinding time of 20 s) and 0−200 μm (grinding time of 30 s), respectively. Those ranges were selected based on the largest size of ground seed that was measured between 0 and 1000 μm and made up more than 60% of the sample [25].
(b) The chemical composition of *Salvia hispanica*, including moisture, ash, crude protein, and fat content, was analyzed using the methodology outlined in the [26] guidelines. The total amount of carbs was determined using the method of difference, as outlined below:
  Carbohydrates% = 100% − the percentages of (moisture + protein + fat + ash)
(c) The determination of amino acids in chia seeds involves the application of ion exchange liquid chromatography, which is a widely used technology for qualitative and quantitative compositional analysis in various fields. The fundamental concept underlying the biochrom systems has been further developed to yield fully automated, rapid, and very sensitive results. The method described by [27] is often known as classical amino acid analysis. The determination of antioxidant activity was conducted using the protocols outlined by [28].
(d) The analyses of vitamins C and E were conducted using high-performance liquid chromatography (HPLC) based on the modified method developed by [29]. The detection of chromatographic studies for vitamin C was performed using an Agilent HPLC system (2000 ECOM, Chrastany u Prahy, CZ 252 19, Czech) with UV detection at a wavelength of 254 nm. In the study conducted by [30], the mobile phase used for the analysis was an analytical column YMC-Triart C18 with dimensions of 150 × 4.6 mm. The mobile phase composition was A/B 33/67, where A consisted of a mixture of 0.1 M potassium acetate and distilled water in a ratio of 50:50 with a pH of 4.9. The flow rate was set at 1 mL/min, and the analysis was performed at the ambient temperature.
(e) The quantification of the total phenolic contents of *Salvia hispanica* was performed through the utilization of high-performance liquid chromatography (HPLC) employing an Agilent 1260 series instrument. The separation procedure was conducted utilizing an Eclipse C18 column with dimensions of 4.6 mm × 250 mm internal diameter and a particle size of 5 μm. The mobile phase was composed of water (A) and a solution of 0.05% trifluoroacetic acid in acetonitrile (B) at a flow rate of 0.9 mL/min. The mobile phase was sequentially programmed in a linear gradient according to the following protocol: In the first minute, the performance was graded at 82% A. From minutes 0 to 5, the performance maintained a grade of 80% A. During the time interval from 5 to 8 min, the performance received a grade of 60% A. Similarly, from 8 to 12 min and 12 to 15 min, the performance maintained a grade of 60% A. However, in the time span from 15 to 16 min, the performance improved and received a grade of 82% A. Finally, from 16 to 20 min, the performance continued to receive a grade of 82% A. The detector operating at several wavelengths was observed at a wavelength of 280 nm. Each of the sample solutions was injected with a volume of 5 μL. The temperature of the column was maintained at 40 °C [31].

### 2.3. Experimental Design

The present investigation was conducted in adherence to ethical guidelines and legislation pertaining to animal testing. The rats were accommodated in wire cages inside an environment where the room temperature was consistently maintained at 25 ± 2 °C. These rats were subjected to standard circumstances that promote their overall well-being and health. During the adaptation period, rats were provided with the previously consumed diet for a duration of one week. In the meantime, an abundant supply of water and meals was made available without restriction. Subsequently, the rats were partitioned into six groups, each consisting of six rats, in the following manner: Group 1 served as the negative control group, denoted as the −Ve control. This group was provided with a basal diet and orally delivered distilled water for a duration of 28 days. The remaining five groups were provided with a standard meal and received intra-peritoneal injections of NaAsO_2_ at a dosage of 4 mg/kg body weight, dissolved in a 0.9% NaCl solution. These injections were administered twice, consecutively, over a period of two days [32]. The rats were categorized into the following groups: Group (2) consisted of a positive control group, denoted as the +Ve control, in which the rats were provided with a baseline diet. In a study by [33], rats from Group (3), Group (4), Group (5), and Group (6) received a basal diet supplemented with 5%, 10%, 15%, and 20% of pulverized chia seeds per 100 g of diet, respectively. Following the conclusion of the experiment, the rats underwent an overnight fasting period before being euthanized. Subsequently, blood samples were obtained from each rat and subjected to centrifugation in order to acquire the serum. The testes and prostate were retrieved, subsequently extracted, and subjected to a cleansing process involving a saline solution. Following this, the specimens were dried using filter paper, and their weights were recorded.

### 2.4. The Assessment of Biological Parameters

Throughout the duration of the trial, which spanned a period of 28 days, the daily feed intake was meticulously documented, while the body weight measurements were taken on a weekly basis. The different diets were tested biologically by measuring the weight of the testicles and prostate (in grams per 100 g of body weight), the amount of food eaten, the percentage of body weight gain, and the feed efficiency ratio, as explained by [34]. By utilizing the subsequent equations:

BWG% = Final weight (g) − Initial weight (g)/Initial weight (g) × 100

and

Feed efficiency ratio (FER) = Weight gain (g)/feed consumed (g)

### 2.5. Biochemical Analysis

Using the established methodologies [35], it was possible to quantify sperm count, sperm motility, progressive motility, and normal form. The serum hormone levels were assessed using the methodology described by [36] for testosterone, and the approach was employed for measuring luteinizing and follicle-stimulating hormones [37]. The serum levels of total T3 (triiodothyronine) and total T4 (thyroxine) were determined using the methodology [38]. The measurement of serum thyroid stimulating hormone (TSH) was conducted using the methodology conducted by [39].

### 2.6. Antioxidant Enzymes and Malondialdehyde in Testes Tissue

The levels of glutathione peroxidase (GPx), catalase (CAT), superoxide dismutase (SOD), and malondialdehyde (MDA) were measured [40]. 

### 2.7. Statistical Analysis

The data obtained from the study were subjected to analysis using the Statistical Package for the Social Sciences (SPSS), specifically Version 25.0. The collected data were provided in the form of a mean standard error (SE). The means of the collected data were compared using a one-way analysis of variance (ANOVA), followed by Duncan’s multiple range tests. The level of statistical significance was established at *p* ≤ 0.05. 

## 3. Result and Discussion

### 3.1. The Phenolic Compounds of Salvia hispanica Seeds Powder (Chia)

Table 1 illustrates the prominent phenolic chemicals found in *Salvia hispanica* seeds powder, namely coffeic acid, ferulic acid, gallic acid, chlorogenic acid, and ellagic acid. The main things that were found in the sample were phenolic and caffeic acids, danshensu, and its byproducts, which include rosmarinic and salvianolic acids. The study’s findings suggest that phytochemicals in *Salvia hispanica* seeds powder, especially gallic acid and protocatechuic acid, may be good for human health by strengthening the body’s antioxidant defense system against free radicals. The findings of our study align with the results reported by [41], who similarly observed the significant antioxidant capacity of these phytochemicals. Moreover, a study conducted by [42] highlights the crucial role of the plant-based mechanism in mitigating lipid oxidation within living tissues. Incorporating plant-based foods into individuals’ diets not only enhances their overall well-being but also reduces the risk of acquiring various illnesses. According to [43], a higher concentration of seeds in food can potentially contribute to the deceleration of the aging process, the mitigation of inflammation, and the alleviation of oxidative stress. These effects are particularly significant as oxidative stress is known to be associated with chronic conditions, such as cardiovascular diseases, arteriosclerosis, cancer, diabetes, cataracts, cognitive impairment, and neurological disorders. Phenolic and polyphenolic compounds, either independently or in conjunction with vitamins such as carotenoids, vitamin E, and vitamin C, possess antioxidant properties that protect human tissues from oxidative stress. Seeds are known to contain a significant amount of antioxidants, particularly polyphenols, caffeic acid, and rosmarinic acid. Research has demonstrated that flavonoids encompass a collection of phytochemicals derived from plants that possess notable attributes such as antioxidant, anti-inflammatory, and anticancer effects. Seeds serve as the primary location of their presence. According to [44], the phenolic chemicals present in *Salvia hispanica* seeds powder, commonly known as chia seeds, have the ability to effectively eliminate free radicals, which are highly reactive molecules capable of causing cellular harm. Simultaneously, they possess the ability to mitigate inflammation, a process that has the potential to inflict harm on cells. Furthermore, it has been demonstrated by [45] that they possess the capability to facilitate the repair of DNA damage. The consumption of chia seeds has the potential to enhance blood circulation to the testes, hence potentially contributing to the enhancement of sperm production [46]. Chia seeds powder have the potential to mitigate the adverse impacts induced by sodium arsenic, such as renal, hepatic, and neurological impairments, as well as testicular damage and compromised sperm production [47].

### 3.2. The Effects of Salvia hispanica Seeds Powder on the Weight of the Prostate and Testes in Male Rats with Induced Testicular Damage from Sodium Arsenite

The findings presented in Table 2 demonstrate a statistically significant reduction in the weight of the prostate and testes in the experimental group (+Ve) compared to the control group (−Ve). Simultaneously, all groups that received treatment exhibited a statistically significant enhancement in average prostate weight and testes weight compared to the control group (+Ve). The groups that received treatment with *Salvia hispanica* seeds powder G3 and G4 (at concentrations of 5% and 10% per 100 g diet) had the most favorable outcomes in comparison to the control group (+Ve). *Salvia hispanica* seeds powder are well recognized as a great dietary resource owing to their substantial nutritional composition, including vital constituents such as fiber, protein, omega-3 fatty acids, and antioxidants [48]. The experimental administration of *Salvia hispanica* seeds powder had a protective effect against testicular and prostate damage induced by arsenic in rats. A reduction in arsenic levels in the testicles and prostate was observed, accompanied by an improvement in the histological structure of the prostate tissue [49]. The utilization of *Salvia hispanica* seeds powder presents additional potential benefits, particularly in relation to the male population. These substances function as a significant source of antioxidants. These chemicals exhibit the ability to protect the human body from harmful effects caused by free radicals. Free radicals, which are chemically unstable substances, possess the capacity to cause damage to biological structures, including DNA, the genetic material [50]. The seeds powder of *Salvia hispanica* are also regarded as a valuable nutritional source of fiber. The ingestion of dietary fiber has the capacity to efficiently sequester arsenic inside the gastrointestinal tract, therefore hindering its absorption into the circulatory system. The seeds powder of *Salvia hispanica* possess a high concentration of omega-3 fatty acids. Omega-3 fatty acids provide anti-inflammatory properties. In the setting of arsenic poisoning, inflammation often presents as a prominent physiological response [51].

### 3.3. The Impact of Salvia hispanica Seeds on Feed Intake (FI), Body Weight Gain Percentage (BWG%), and Feed Efficiency Ratio (FER) in Male Rats with Experimentally Induced Testicular Toxicity Caused by NaAsO_2_

The results reported in Table 3 illustrate the impact of *Salvia hispanica* seeds powder on variables such as (FI), (BWG%), and (FER). The findings indicated a notable reduction in feed intake, body weight increase, and feed efficiency ratio in the sodium arsenite control group (+Ve) when compared to the control group (−Ve). The experimental groups that received *Salvia hispanica* seeds powder exhibited a noteworthy augmentation in their (FI), (BWG%), and (FER) in comparison to the control group (+Ve). The groups that exhibited the most favorable outcomes were those that received treatments with *Salvia hispanica* seeds powder G3 and G4, with concentrations of 5% and 10% per 100 g diet, respectively, in comparison to the control group (+Ve). Sodium arsenic poisoning is a medically significant illness that can give rise to several health concerns, including decreased body weight, reduced appetite, and impaired digestive function [51]. Previous research has shown that the use of *Salvia hispanica* seeds powder can lead to increases in body weight and body composition among rats affected by salt and arsenic poisoning. The inclusion of observable variables such as an augmentation in feed intake and an enhancement in feed efficiency ratio [22]. In a study conducted by [52], the consumption of *Salvia hispanica* seeds powder has been associated with increased feed intake and an enhanced feed efficiency ratio. These effects can be due to the significant content of dietary fiber, protein, and essential fatty acids present in these seeds. The presence of sodium arsenic has been found to potentially lead to the occurrence of inflammation and oxidative stress in the human body. The seeds powder of *Salvia hispanica* have anti-inflammatory and antioxidant properties, which may potentially aid in reducing inflammation and oxidative stress in rats suffering from sodium arsenic poisoning [53]. The promise of chia seeds to improve gut health lies in their capacity to promote the growth of beneficial gut bacteria. The maintenance of an optimum gut microbiota is of utmost importance for the efficient process of digestion and the subsequent absorption of essential nutrients.

### 3.4. The Effect of Salvia hispanica Seeds Powder on Sperm Parameters in Rats Suffering from NaAsO_2_-Induced Testicular Damage

When sodium arsenite was given, there was a significant drop in the number of sperm, their ability to move, their ability to move over time, and their normal shape (*p* < 0.05) compared to the control group (−Ve). Compared to the control group (+Ve), giving *Salvia hispanica* seeds powder to groups of rats (3, 4, 5, and 6 individuals) greatly increased the number of sperm, their ability to move, their ability to move over time, and their normal shape. The groups that received treatment with *Salvia hispanica* seeds powder G3 and G4 (at concentrations of 5% and 10% per 100 g diet) exhibited the most favorable outcomes in comparison to the control group (+Ve), as indicated in Table 4. The decline in male fertility has been a notable concern within the realm of public health in recent times, a trend that has corresponded with an increase in the extent of exposure to detrimental environmental pollutants [54]. The harmful effects on male fertility seem to be attributed to the presence of hazardous elements such as arsenic (As) and lead (Pb), which progressively compromise the quality of semen [55]. The observed association between sperm count, sperm motility, and sperm morphology may be explained by the influence of arsenic exposure on the increased production of reactive oxygen species. Consequently, this phenomenon exerts an influence on the spermatogenesis process occurring in the testes as well as the subsequent storage of spermatozoa within the seminiferous tubules. The harmful effects of arsenic are manifested by the stimulation of reactive oxygen species (ROS) generation during redox cycling and metabolic activation mechanisms, resulting in tissue impairment [56]. The deleterious impacts of free radicals on biomembranes are apparent through the observed increase in lipid peroxidation, oxidation of nucleic acids, and degradation of proteins. As a result, these processes give rise to a degraded state of cellular integrity and decreased functionality [57]. Researchers have seen that compounds containing arsenic can damage DNA. This damage includes the creation of free radicals, which lower the function of sperm. A significant decrease in the diameter of the seminiferous tubules and a decline in sperm quality following exposure to arsenic [58]. Arsenic is a well-established metalloid that functions as a thiol inhibitor and plays a vital role in maintaining the viability and integrity of sperm. The potential cause for the decrease in sperm motility found in this study may be associated with the accumulation of arsenic within the epididymis, which serves as the location for sperm maturation and the acquisition of motility. Furthermore, the decrease observed in rats exposed to arsenic may be attributed to the diminished availability of testosterone [59]. The seeds powder of *Salvia hispanica* contain a significant amount of antioxidants, particularly polyphenols, which have the capacity to alleviate the adverse impacts of oxidative stress caused by heavy metals like lead and arsenic [60]. The adverse impacts of oxidative stress on the well-being of sperm span multiple dimensions, including but not limited to sperm count, motility, and DNA integrity. The potential protective effect of *Salvia hispanica* seeds powder on sperm quality can be linked to their capacity to mitigate oxidative stress. *Salvia hispanica* seeds powder is rich in omega-3 fatty acids, particularly alpha-linolenic acid (ALA) [61]. The significance of omega-3 fatty acids in relation to reproductive health is noteworthy due to their association with improved sperm parameters in certain research studies. These compounds have the capacity to enhance the quantity, motility, and morphology of sperm [62]. The seeds powder of *Salvia hispanica* are abundant in nutrients and encompass essential vitamins, minerals, and proteins. Adequate nutrition plays a vital role in preserving optimal health, including various dimensions, including reproductive function [47].

### 3.5. The Impact of Salvia hispanica Seeds Powder on Testicular Hormone Levels in Male Rats with Experimentally Produced Testicular Damage Caused by NaAsO_2_

Table 5 presents the plasma levels of (FSH), (LH), and testosterone in various groups under investigation. A large drop (*p* < 0.05) was seen in the levels of (FSH), (LH), and testosterone in the rats that were given arsenic (+Ve) compared to the rats in the control group (−Ve). The levels of (FSH), (LH), and testosterone hormone were statistically significantly higher in the other groups than in the control (+Ve). The groups that showed the most favorable outcome were those who received *Salvia hispanica* seeds powder G3 and G4 (at concentrations of 5% and 10% per 100 g of food). These groups exhibited results that were closest to those of the control group (−Ve). It was found that adding sodium arsenite to the body significantly lowered levels of testosterone and (LH). It also decreased the number and movement of sperm. Also, Ref. [4] Zubair et al. found that arsenic stops the release of (FSH) and (LH), which lowers the production of testosterone [4]. Arsenic demonstrates a significant affinity for the binding of glutathione, a molecule that possesses sulfhydryl groups. The interplay between arsenic and the sulfhydryl groups present in glutathione leads to a decrease in the amount of glutathione, thereby impeding the activity of glutathione reductase. As a result, this particular procedure leads to an excessive generation of reactive oxygen species inside the testicular tissue [63]. The excessive production of reactive oxygen species (ROS) induces lipid peroxidation in cellular membranes, hence causing cellular dysfunction [64]. The increased production of free radicals negatively impacts the effectiveness of the antioxidant defense system, resulting in tissue damage. The current study shows that exposure to arsenic (AS) led to a significant increase in the levels of testicular (MDA) [65]. The antioxidant properties of *Salvia hispanica* seeds powder possess the capacity to attenuate oxidative processes and the production of free radicals within the human body. The seeds powder of *Salvia hispanica* contain a diverse range of polyphenolic compounds, such as flavonoids and phenolic acids, that have antioxidant characteristics [46]. Polyphenols have been found to have the ability to efficiently remove free radicals and mitigate their adverse effects. Through active participation in this particular activity, individuals make a valuable contribution towards the mitigation of oxidative harm to cellular and tissue structures [66]. *Salvia hispanica* seeds powder is widely recognized as a significant dietary source of vitamin E, a fat-soluble antioxidant. The role of vitamin E is essential in protecting cellular membranes against oxidative damage. It actively contributes to the prevention of lipid peroxide formation and the adverse effects associated with oxidation processes [67]. The seeds powder of *Salvia hispanica* contain significant amounts of essential minerals, such as selenium and zinc, which function as cofactors for antioxidant enzymes in the human body. Enzymes like (SOD) and glutathione peroxidase play a big role in protecting cells from oxidative damage and getting rid of free radicals [25]. The seeds powder of *Salvia hispanica* possess a high content of dietary fiber. Fiber might indirectly contribute to the enhancement of antioxidant defense systems by promoting intestinal health and inhibiting the accumulation of harmful substances in the body. The efficient operation of the digestive system is of utmost importance in the removal of harmful substances and the reduction of oxidative damage to cellular components [68].

### 3.6. Effects of Salvia hispanica Seeds Powder on Thyroid Hormone Levels in Male Rats with NaAsO_2_-Induced Testicular Damage

In terms of thyroid hormones, Table 6 shows that the levels of T3, T4, and TSH were significantly lower (*p* < 0.05) in rats that were given sodium arsenate. When T3, T4, and TSH levels were analyzed, it was found that all experimental groups had a statistically significant rise compared to the control group (+Ve). *Salvia hispanica* seeds powder G1 and G4 were given to the experimental group at concentrations of 5% and 10% per 100 g of diet. This group had very good results, as their recorded values were very close to those of the control group (−Ve). The impact of arsenic on thyroid hormones is significant, as it predominantly affects the thyroid gland and its associated physiological processes, leading to alterations in hormone levels and functioning within the body. Arsenic, a metalloid with hazardous properties, possesses the capacity to interfere with the normal physiological processes of the thyroid gland [69]. The compound possesses the capacity to hinder the assimilation of iodine, a vital ingredient necessary for the synthesis of thyroid hormones. The sodium-iodide symporter (NIS) protein is needed for iodine uptake to work well in the thyroid gland [70]. The NIS protein can undergo competitive inhibition when exposed to arsenic, leading to a reduction in iodine uptake and interference with the synthesis of thyroid hormones. Moreover, it has been found that arsenic possesses the capacity to interfere with the intricate process of synthesizing thyroid hormones within the thyroid gland [71]. The chemical in question has the capacity to disrupt the enzymatic mechanisms involved in the conversion of iodide to iodine, as well as the subsequent conversion of iodine into thyroid hormones, namely (T4) and (T3) [72]. The interference stated above has the potential to lead to a decrease in the production of thyroid hormones and an imbalance in their levels. The existence of arsenic possesses the capacity to influence the regulation of thyroid hormones through many methods [73]. It can change the hypothalamic-pituitary-thyroid (HPT) axis, which controls how much (TSH) is released from the pituitary gland and how it is affected by feedback. Arsenic exposure may change the production and sensitivity of (TSH), which can throw off the balance of thyroid hormones as a whole [74]. *Salvia hispanica* seeds powder possess a substantial quantity of antioxidants, comprising polyphenols and omega-3 fatty acids. Antioxidants have the capacity to alleviate the adverse consequences of oxidative stress caused by exposure to arsenic. The introduction of arsenic can potentially lead to the manifestation of thyroid dysfunction through the creation of oxidative stress [75]. The seeds powder of *Salvia hispanica* possess the capability to alleviate oxidative stress, potentially protecting the thyroid gland from damage and facilitating its normal physiological functions. The seeds powder of *Salvia hispanica* are considered to be a rich nutritional resource because of their significant concentration of essential components, including iodine [76]. Adequate iodine levels are necessary for the proper synthesis of thyroid hormones. The seeds powder of *Salvia hispanica* possess the capability to mitigate the deficiency of iodine resulting from arsenic poisoning and facilitate the production of thyroid hormones by acting as a dietary source of iodine [77].

### 3.7. The Effects of Salvia hispanica Seeds Powder on Antioxidant Enzymes and Malondialdehyde Levels in Male Rats with Experimentally Induced Testicular Damage from NaAsO_2_

The main focus of the study was considering (GPx), (SOD), and (CAT) as important, naturally occurring antioxidant enzymes in the testicular tissue of the rats that were being studied. The data in Table 7 clearly show that the rats that were given sodium arsenate had significantly higher levels of MDA (*p* < 0.05), but significantly lower levels of GPx, SOD, and CAT (*p* < 0.05). The results of the experiment show that the treatment groups had significantly higher levels of GPx, SOD, and CAT compared to the control group (+Ve). The levels of (MDA) were statistically significantly lower in all experimental groups compared to the control group (+Ve). The experimental group that was administered *Salvia hispanica* seeds powder G4 (10%/100 g diet) exhibited the most advantageous result, as the recorded values closely mirrored those of the control group (−Ve). When sodium arsenite is present in the body, it raises the levels of lipid peroxides, which hurts cellular membranes and other important parts [78]. The seeds powder of *Salvia hispanica* possess significant quantities of antioxidants and polyphenols, which have the potential to mitigate the adverse impacts of free radicals. A variety of bioactive compounds, such as chlorogenic acid, caffeic acid, myricetin, quercetin, and kaempferol, possess anti-inflammatory and antioxidant characteristics [46]. Flavonoids, including anthocyanins and quercetin, provide beneficial characteristics that contribute to the promotion of health. These compounds exhibit the ability to counteract the effects of free radicals and restrict the production of inflammatory molecules. The seeds powder of *Salvia hispanica* has a high concentration of omega-3 fatty acids, which possess qualities that can mitigate inflammation and act as antioxidants [79]. These fatty acids possess the ability to mitigate the detrimental impacts of free radicals and hinder the production of inflammatory compounds. The ingestion of *Salvia hispanica* seeds has the potential to improve treatment results in cases of arsenic poisoning [80,81,82,83]. This improvement is attributed to the seeds’ ability to increase the production of oxidation enzymes, reduce the amount of lipid peroxides, and protect cells against the detrimental effects caused by free radicals. 

### 3.8. Histopathology Examination

**Figure 1:**
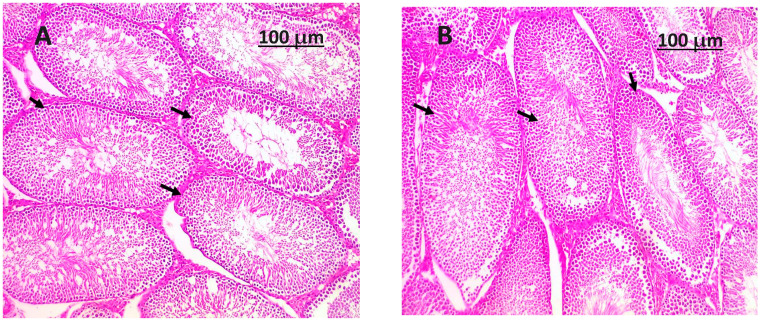
(**A**,**B**); If you look closely at the H&E-stained section of (a normal testis) from the negative control group (G1), which was only fed a baseline diet, you can see seminiferous tubules (shown by black arrows). These tubules were observed to be surrounded by layers of spermatogenic cells and contain an abundance of spermatozoa.

**Figure 2:**
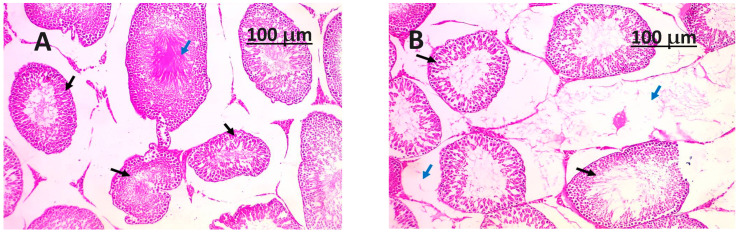
(**A**,**B**) the positive control group (G2) (testicular toxicity), which was injected with sodium arsenite, had a section of the testis that was stained with H&E. This section showed that many of the seminiferous tubules were damaged, as shown by the blue arrows. Additionally, there were disorganized tubules (depicted by black arrows) with stopped sperm production.

A close look at the testicle in its normal state through histology (shown in Figure 1) shows that the seminiferous tubules are still in good shape, showing that the germinal epithelium and Sertoli cells are still there. The interstitial tissue is comprised of Leydig cells, which are accountable for the synthesis and secretion of testosterone. The presence of an intact rete testis and the provision of an adequate blood supply are essential factors that contribute to the proper functioning of the organ. The *Salvia hispanica* seeds powder plays a crucial role in maintaining structural integrity and safeguarding the testicle. In general, the morphological and histological characteristics that were observed align with a state of sound and typical testicular structure. Simultaneously, the histological analysis of the testicle afflicted with testicular toxicity (as depicted in Figure 2) generated by sodium arsenic demonstrates noteworthy pathological alterations. The observed phenomena encompass degeneration, disruption, and loss of the seminiferous tubular structure, modifications in interstitial tissue, vascular impairment, as well as cellular and tissue irregularities. The observed alterations combined suggest the adverse impacts of sodium arsenic on the tissue of the testes. The histological changes found in this study are in line with testicular toxicity, which can have adverse impacts on both testicular function and reproductive health.

**Figure 3:**
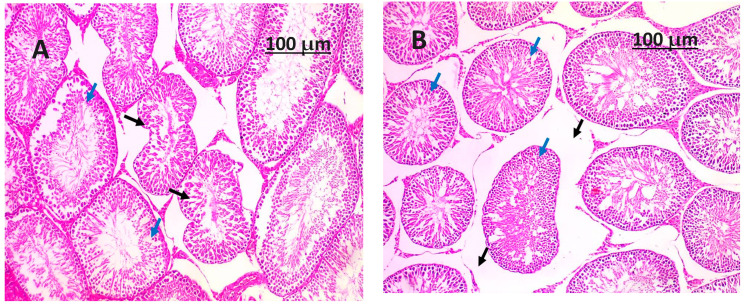
(**A**,**B**) show histological pictures of the H&E-stained testis section of rats fed a base diet with 5% and 10% of pulverized chia seeds per 100 g of food, respectively. The rats were given a treatment. The histological examination revealed the presence of well-formed tubules (shown by blue arrows) in the (**A**) sample, exhibiting complete spermatogenesis. Notably, a number of these tubules (represented by black arrows) showed no signs of destruction. The histological examination revealed the presence of well-developed tubules (shown by blue arrows) in the (**B**) sample, exhibiting spermatogenesis in numerous tubules without any signs of degeneration. However, there was a noticeable broad separation observed between the tubules (indicated by black arrows).

**Figure 4:**
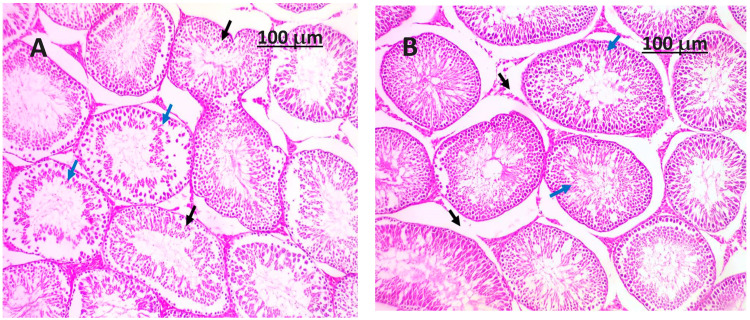
(**A**,**B**) show histological pictures of the H&E-stained testis section of testis from rats fed a diet with 15% and 20% of ground chia seeds per 100 g diet, respectively, showed that the (**A**,**B**) samples had focally disorganized tubules, which are shown by black arrows. However, there was evidence of improved spermatogenesis in numerous tubules (indicated by blue arrows). The image demonstrates the progression of (**B**) spermatogenesis up until the generation of spermatids (shown by blue arrows), which are observed within the context of adjacent tubules exhibiting enhanced spermatogenesis (indicated by black arrows).

The histopathological assessment of the experimental groups administered with (*Salvia hispanica*) chia seeds powder (as depicted in Figure 3 and Figure 4) reveals there may still be observable differences in size and weight between the damaged testicle and a healthy one, but the introduction of chia seeds as an intervention has the ability to reduce the degree of size reduction and weight loss associated with poisoning. The testicular surface may still display certain abnormalities or discoloration, although the extent of these alterations is more severe when compared to a testicle solely impacted by poisoning, as depicted in Figure 2. The seminiferous tubules may exhibit different levels of preservation and cellular arrangement in comparison to a testicle impacted solely by toxicity, as depicted in Figure 2. The potential benefits of chia seed intervention include the preservation of the germinal epithelium’s integrity, the reduction of degeneration, and the limitation of cellular structure loss within the tubules, as depicted in Figure 3 and Figure 4. The interstitial tissue exhibits a diminished presence of inflammatory markers, edema, or fibrotic alterations (as depicted in Figure 3 and Figure 4) in contrast to a testicle impacted solely by toxicity (as depicted in Figure 2). The implementation of chia seed interventions has the ability to enhance the equilibrium of the interstitial environment, thus mitigating the deleterious effects on the Leydig cells, which play a crucial role in the synthesis of testosterone. The presence of blood vessels in the testicle may suggest a lower degree of congestion or vascular impairment (as depicted in Figure 3 and Figure 4) in comparison to a testicle only damaged by toxicity (as depicted in Figure 2). The implementation of chia seeds powder interventions has the potential to contribute to the preservation of vascular integrity and mitigate the extent of endothelial cell abnormalities or thrombotic transformations. The introduction of (*Salvia hispanica*) chia seeds powder interventions may potentially lead to a decrease in apoptotic processes and a reduction in inflammation in the testicular tissue. This phenomenon has the potential to enhance the preservation of biological constituents and mitigate the infiltration of immune cells. 

## 4. Conclusions

Chia seeds, formally known as *Salvia hispanica*, are categorized as oilseeds and are widely acknowledged as pseudo-cereals. These seeds contain a wide variety of nutrients, including both macro- and micronutrients, as well as qualities that promote good health. Consequently, they might be classified as a kind of nutraceutical food. The seeds powder act as a very effective storage medium for phenolic compounds, including rosmarinic acid, caffeic acid, protocatechuic acids, quercetin, and myricetin. The research indicates that the bioactive compounds found in *Salvia hispanica* (Chia) seeds powder have the potential to mitigate inflammation. This research might contribute to the advancement of dietary interventions for those affected by salt and arsenic poisoning. *Salvia hispanica* is a plant species renowned for its antioxidative properties. Evidence demonstrates its ability to eliminate free radicals, sustain the functionality of antioxidant enzymes, and reduce the presence of inflammatory mediators. Therefore, it has the capacity to be used as a therapeutic agent to alleviate testicular damage and dysfunction caused by arsenic exposure. Recent study findings suggest that chia seeds powder possess several health-enhancing characteristics, especially testicular toxicity. These seeds have a favorable impact on enhancing the blood lipid profile. The experiments validated the hypotensive, hypoglycemic, antibacterial, and immunostimulatory properties. Chia seeds powder may be used in food technology as a replacement for emulsifiers and stabilizers due to their ability to absorb water and produce gels. To summarize, chia seeds (*Salvia hispanica*) are a rich natural resource with versatile technical and health-enhancing features that may be extensively used in the food industry.

## Figures and Tables

**Table 1:** Phenolic compounds in *Salvia hispanica* seeds powder (Chia).

*Salvia hispanica* Seeds Powder (Chia Seeds)	
Phenolic Compounds	Conc. (µg/g)
Gallic acid	833.35 ± 15.10
Chlorogenic acid	1717.79 ± 20.42
Catechin	3918.61 ± 12.65
Coffeic acid	9596.08 ± 18.32
Rutin	828.77 ± 11.45
Ellagic acid	2028.77 ± 16.27
Vanillin	199.55 ± 28.10
Ferulic acid	2085.90 ± 24.11
Naringenin	1449.25 ± 9.05
Daidzein	509.53 ± 23.08
Querectin	1584.25 ± 35.16
Cinnamic acid	9.94 ± 0.04
Apigenin	256.69 ± 10.27
Hesperetin	359.28 ± 18.23

The results are expressed as mean ± SE

**Table 2:** Effect of *Salvia hispanica* seeds powder on weight of prostate and testes with induced testicular toxicity induced by NaAsO_2_ in male rats.

Groups	Parameters	
	Prostate Weight (g/100 g b.w)	Testes Weight (g/100 g b.w)
	(Mean ± SE)	(Mean ± SE)
G1 (−Ve)	0.60 ± 0.012 ^a^	2.34 ± 0.015 ^a^
G2 (+Ve)	0.32 ± 0.014 ^d^	1.62 ± 0.012 ^d^
G3 (5%/100 g diet)	0.56 ± 0.012 ^b^	2.24 ± 0.014 ^b^
G4 (10%/100 g diet)	0.59 ± 0.008 ^a^	2.28 ± 0.008 ^b^
G5 (15%/100 g diet)	0.55 ± 0.006 ^b^	2.16 ± 0.011 ^bc^
G6 (20%/100 g diet)	0.52 ± 0.014 ^c^	2.02 ± 0.014 ^c^

Data are presented as Mean ± SE. Values bearing dissimilar alphabets as superscripts significant at *p* ≤ 0.05, n = (6 rats).

**Table 3:** Effect of *Salvia hispanica* seeds powder on (FI), (BWG%) and (FER) with induced testicular toxicity induced by NaAsO_2_ in male rats.

Groups	Parameters		
	FI (g/28 days)	BWG%	FER
	Mean ± SE	Mean ± SE	Mean ± SE
G1 (−Ve)	294.99 ± 0.86 ^a^	28.56 ± 0.88 ^a^	0.12 ± 0.005 ^a^
G2 (+Ve)	238.21 ± 1.17 ^f^	18.18 ± 0.84 ^d^	0.06 ± 0.007 ^d^
G3 (5%/100 g diet)	283.75 ± 1.75 ^c^	27.23 ± 1.15 ^ab^	0.11 ± 0.009 ^b^
G4 (10%/100 g diet)	288.29 ± 1.47 ^b^	28.85 ± 0.87 ^b^	0.10 ± 0.005 ^b^
G5 (15%/100 g diet)	275.23 ± 1.16 ^d^	23.95 ± 0.89 ^bc^	0.08 ± 0.007 ^bc^
G6 (20%/100 g diet)	262.44 ± 1.20 ^e^	21.99 ± 0.86 ^c^	0.07 ± 0.008 ^c^

Data are presented as Mean ± SE. Values bearing dissimilar alphabets as superscripts significant at *p* ≤ 0.05. n = (6 rats). FI = Feed intake, BWG% = Body weight gain percentage, and FER = Feed efficiency ratio.

**Table 4:** Effect of *Salvia hispanica* seeds powder on sperm parameters in rats with induced testicular toxicity induced by NaAsO_2_ in male rats.

Groups	Parameters			
	Sperm Count (×10^6^/mL)	Sperm Motility (%)	Progressive Motility (%)	Normal Form (%)
	Mean ± SE	Mean ± SE	Mean ± SE	Mean ± SE
G1 (−Ve)	67.42 ± 1.01 ^a^	78.34 ± 2.41 ^a^	65.00 ± 1.60 ^a^	75.06 ± 2.11 ^a^
G2 (+Ve)	32.15 ± 2.45 ^e^	36.63 ± 2.02 ^e^	31.14 ± 1.25 ^f^	37.03 ± 1.04 ^f^
G3 (5%/100 g diet)	62.92 ± 3.10 ^c^	70.61 ± 2.03 ^b^	58.36 ± 1.50 ^c^	68.42 ± 2.34 ^c^
G4 (10%/100 g diet)	65.67 ± 2.41 ^b^	74.35 ± 2.06 ^ab^	62.00 ± 2.14 ^b^	73.06 ± 2.11 ^b^
G5 (15%/100 g diet)	54.10 ± 3.16 ^cd^	58.72 ± 1.56 ^c^	46.00 ± 2.08 ^d^	58.14 ± 1.63 ^d^
G6 (20%/100 g diet)	51.30 ± 3.17 ^d^	54.64 ± 2.31 ^d^	40.23 ± 2.16 ^e^	51.23 ± 1.13 ^e^

Data are presented as Mean ± SE. Values bearing dissimilar alphabets as superscripts significant at *p* ≤ 0.05. n = (6 rats)

**Table 5:** Effect of *Salvia hispanica* seeds powder on testes hormone’s in rats with induced testicular toxicity induced by NaAsO_2_ in male rats.

Groups	Parameters		
	FSH (ng/mL)	LH (ng/mL)	Testosterone H. (ng/mL)
	Mean ± SE	Mean ± SE	Mean ± SE
G1 (−Ve)	1.38 ± 0.02 ^a^	1.89 ± 0.05 ^a^	3.65 ± 0.05 ^a^
G2 (+Ve)	0.18 ± 0.01 ^c^	0.39 ± 0.03 ^f^	1.16 ± 0.01 ^f^
G3 (5%/100 g diet)	1.17 ± 0.03 ^bc^	1.47 ± 0.02 ^c^	2.92 ± 0.02 ^c^
G4 (10%/100 g diet)	1.22 ± 0.02 ^b^	1.55 ± 0.04 ^b^	3.05 ± 0.03 ^b^
G5 (15%/100 g diet)	1.01 ± 0.02 ^d^	1.31 ± 0.03 ^d^	2.63 ± 0.04 ^d^
G6 (20%/100 g diet)	0.85 ± 0.04 ^e^	1.20 ± 0.01 ^e^	2.40 ± 0.03 ^e^

Data are presented as Mean ± SE. Values bearing dissimilar alphabets as superscripts significant at *p* ≤ 0.05. n = (6 rats). FSH = Follicle-stimulating hormone, and LH = luteinizing hormone.

**Table 6:** Effect of *Salvia hispanica* seeds powder on thyroid hormones in rats with induced testicular toxicity induced by NaAsO_2_ in male rats.

Groups	Parameters		
	T3 (ng/mL)	T4 (ug/dL)	TSH (µlU/mL)
	Mean ± SE	Mean ± SE	Mean ± SE
G1 (−Ve)	0.86 ± 0.04 ^a^	6.80 ± 0.48 ^a^	0.0015 ± 0.0001 ^a^
G2 (+Ve)	0.63 ± 0.02 ^e^	4.51 ± 0.17 ^f^	0.0009 ± 0.0000 ^f^
G3 (5%/100 g diet)	0.81 ± 0.01 ^b^	6.63 ± 0.03 ^c^	0.0012 ± 0.0002 ^c^
G4 (10%/100 g diet)	0.84 ± 0.03 ^ab^	6.76 ± 0.20 ^b^	0.0014 ± 0.0002 ^b^
G5 (15%/100 g diet)	0.73 ± 0.05 ^c^	6.12 ± 0.27 ^d^	0.0011 ± 0.0001 ^d^
G6 (20%/100 g diet)	0.70 ± 0.02 ^d^	5.65 ± 0.18 ^e^	0.0010 ± 0.0001 ^e^

Data are presented as Mean ± SE. Values bearing dissimilar alphabets as superscripts significant at *p* ≤ 0.05., n = (6 rats). T3 = Triiodothyronine, T4 = Thyroxine, and TSH = Thyroid-stimulating hormone.

**Table 7:** Effect of *Salvia hispanica* seeds powder on antioxidant enzymes and malondialdehyde in rats with induced testicular toxicity induced by NaAsO_2_ in male rats.

Groups	Parameters			
	GP_X_ (ng/mg)	SOD (U/min/mg )	CAT (U/min/mg )	MDA (nMol/mg )
	Mean ± SE	Mean ± SE	Mean ± SE	Mean ± SE
G1 (−Ve)	0.54 ± 0.02 ^a^	0.37 ± 0.01 ^a^	0.35 ± 0.02 ^a^	0.10 ± 0.01 ^e^
G2 (+Ve)	0.17 ± 0.01 ^f^	0.12 ± 0.02 ^e^	0.14 ± 0.01 ^f^	0.28 ± 0.04 ^a^
G3 (5%/100 g diet)	0.48 ± 0.03 ^c^	0.33 ± 0.004 ^b^	0.30 ± 0.04 ^c^	0.15 ± 0.02 ^d^
G4 (10%/100 g diet)	0.50 ± 0.04 ^b^	0.35 ± 0.01 ^ab^	0.33 ± 0.02 ^b^	0.14 ± 0.03 ^d^
G5 (15%/100 g diet)	0.35 ± 0.02 ^d^	0.31 ± 0.02 ^c^	0.29 ± 0.03 ^d^	0.20 ± 0.01 ^c^
G6 (20%/100 g diet)	0.24 ± 0.05 ^e^	0.27 ± 0.03 ^d^	0.24 ± 0.01 ^e^	0.24 ± 0.02 ^b^

Data are presented as Mean ± SE. Values bearing dissimilar alphabets as superscripts significant at *p* ≤ 0.05., n = (6 rats). GP_X_ = Glutathione peroxidase, SOD = Superoxide dismutase, CAT = Catalase, and MDA = Malondialdehyde.

## Data Availability

Data is contained within the article.
